# Digital DC-Reconstruction of AC-Coupled Electrophysiological Signals with a Single Inverting Filter

**DOI:** 10.1371/journal.pone.0150207

**Published:** 2016-03-03

**Authors:** Roger Abächerli, Jonas Isaksen, Ramun Schmid, Remo Leber, Hans-Jakob Schmid, Gianluca Generali

**Affiliations:** 1 Signal Processing and Research, Research and Development, Schiller AG, Baar, Switzerland; 2 Cardiovascular Research Institute, University Hospital Basel, Basel, Switzerland; 3 Bern University of Applied Sciences, Bern, Switzerland; 4 Laboratory of Experimental Cardiology, University of Copenhagen, Copenhagen, Denmark; Shenzhen institutes of advanced technology, CHINA

## Abstract

Since the introduction of digital electrocardiographs, high-pass filters have been necessary for successful analog-to-digital conversion with a reasonable amplitude resolution. On the other hand, such high-pass filters may distort the diagnostically significant ST-segment of the ECG, which can result in a misleading diagnosis. We present an inverting filter that successfully undoes the effects of a 0.05 Hz single pole high-pass filter. The inverting filter has been tested on more than 1600 clinical ECGs with one-minute durations and produces a negligible mean RMS-error of 3.1*10^−8^ LSB. Alternative, less strong inverting filters have also been tested, as have different applications of the filters with respect to rounding of the signals after filtering. A design scheme for the alternative inverting filters has been suggested, based on the maximum strength of the filter. With the use of the suggested filters, it is possible to recover the original DC-coupled ECGs from AC-coupled ECGs, at least when a 0.05 Hz first order digital single pole high-pass filter is used for the AC-coupling.

## Introduction

The AC-coupling used in electrocardiographic (ECG) recorders is known to produce distortions in the diagnostically important ST-segment of the ECG waveform [1]. Since the introduction of digital electrocardiographs, a high-pass filter (HPF) has been necessary to limit the signal to the desired range, for instance ±5 mV, specified by the analog-to-digital converter (ADC). The proposed solution of using a first order 0.05 Hz high-pass filter was accepted in the AHA guidelines published in 1967 [2]. Both the current American [3] and European [4] standards still suggest the use of the first order high-pass filter with a cut-off frequency of 0.05 Hz, or an equivalent linear-phase filter. Such filters have become the industrial standard.

Many alternatives to *digital* 0.05 Hz filtering have been suggested. Often, the baseline is estimated and then subtracted from the original signal. Meyer *et al*. [5] suggested a state-space estimation of the baseline using cubic splines. Van Alsté *et al*. [6] suggested a FIR-filter that combined powerline filtering with baseline filtering. Different types of adaptive filtering, including the use of the wavelet transform, were suggested by a number of researchers, including Laguna *et al*., Wang *et al*., and Park *et al*. [7–9]. Chu and Delp [10] used morphological operators to remove both baseline wander and high-frequency noise. Blanco-Velasco *et al*. [11] suggested combining EMD [12] and a bank of filters for general ECG denoising. The EMD method was combined with a mean-median filter by Xin *et al*. [13]. The method of quadratic variation reduction has been suggested by Fasano *et al*. [14]. As opposed to the first order high-pass filter, these alternatives require at least a relatively long delay or they must be implemented as off-line solutions. Furthermore, they all require higher performance as compared to a first order IIR-filter.

With the development of cheaper ADCs with more bits in recent years, it is possible to use the DC-coupling that was suggested as ideal by Berson *et al*. [1], without compromising signal resolution [15].

It is therefore now possible to acquire an ECG signal at 1 μV/bit with full dynamics of ±500 mV without any remaining gain and phase distortions due to high-pass filter effects, as the analog filter is left out. The only limiting factor that remains is the anti-aliasing filter needed prior to the analog-to-digital conversion in order to avoid aliasing [16].

The question that one may ask is to what degree the AC-coupled ECG signals can be compared to the DC-coupled ones, and if one can be converted into the other. The conversion of a DC-coupled signal into an AC-coupled signal is straightforward and gives some insight into how the AC-coupling distorts the ECG signal morphology. It is well-known that the 0.05 Hz HPF may distort the ST-segment [1], and this may generate a misleading diagnosis. If we can convert the existing digital ECG signal acquired with an AC-coupling into DC-coupled signals, the degree of morphological distortions and their clinical relevance can be assessed in full detail.

In this work, we present a simple so-called inverse digital filter that is capable of restoring original DC-coupled signals after their passing through a digital first order single pole high-pass filter–simulating the analog AC-coupling–with an absolute error well below 0.5 Least Significant Bit (LSB) for the waveform. Only the original DC-level cannot be recovered. We furthermore specify how to design the filter based on the maximal amplification (which is found at zero frequency) tolerated for the inverse filter.

## Materials and Methods

### Inverse filter design

A first order digital high-pass filter (HPF), constrained to have a unity gain in the pass-band, *i*.*e*. H(z = -1) = 1, is given by (1) [17]. The filter has a zero at z = 1 and a pole at z = β that controls the cut-off frequency. Further details are given in appendix A.

$$H_{1}\left(z\right)=\frac{\beta +1}{2}\frac{z-1}{z-\beta }$$ (1)

Most effects caused by such a system can be reversed by the use of an “inverse high-pass filter” (iHPF), with some exceptions: firstly, the DC-offset present in a signal cannot be recovered since this frequency component is “infinitely suppressed”.

Secondly, the rounding operation (quantization), which may be performed after the analog or digital high-pass filtering, adds uncorrectable errors: by rounding, a white noise error signal is added to the physiological signal [18]. Further, all signal content with an amplitude reduced to less than 1 LSB by the high-pass filter is lost. The lower the frequency–or the higher the cut-off frequency of the filter–the higher amplitudes can be masked via this effect. However, for a signal to fully contain very low frequency content, the duration of the signal must be sufficiently long.

The iHPF is constructed in such a way that the product of both filters is unity, *i*.*e*. simply by inverting the transfer function of the HPF [19]:

$$H_{2}\left(z\right)=\frac{1}{H_{1}\left(z\right)}=\frac{2}{\beta +1}\frac{z-\beta }{z-1}$$ (2)

What may be a problem is that the second filter now has a pole at z = 1 (DC), *i*.*e*. the filter is not stable since the pole is not within the unit circle but exactly on the unit circle. Equivalently, the amplification at DC is infinite.

This problem can be overcome by shifting the pole slightly towards the origin, so that the pole lies within the unit circle. The resulting filter (HPF followed by iHPF) is then effectively a new first order high-pass filter with a different cut-off frequency than originally, because the HPF-pole and iHPF-zero cancel each other out. Assuming the iHPF-pole is not shifted past the HPF-zero, the resulting cut-off frequency is lowered. Using this specification, the stable iHPF takes the form of (3), whereby β originates from the HPF and c remains to be specified.

$$H_{3}\left(z\right)=\frac{c+1}{2}\frac{z-1}{z-c}\frac{2}{\beta +1}\frac{z-\beta }{z-1}=\frac{c+1}{\beta +1}\frac{z-\beta }{z-c}$$ (3)

A filter of this type obviously has its largest gain at DC following the description above. We denote this maximal gain as M and the iHPF can be designed based on this maximal gain (4). Note that c tends to 1 as M tends to infinity, and in this case, (3) reduces to (2). Practical values for M are suggested later.

$$c=1-2\frac{1-\beta }{M\left(\beta +1\right)+1-\beta },\left|\beta \right|<1$$ (4)

**Fig 1:**
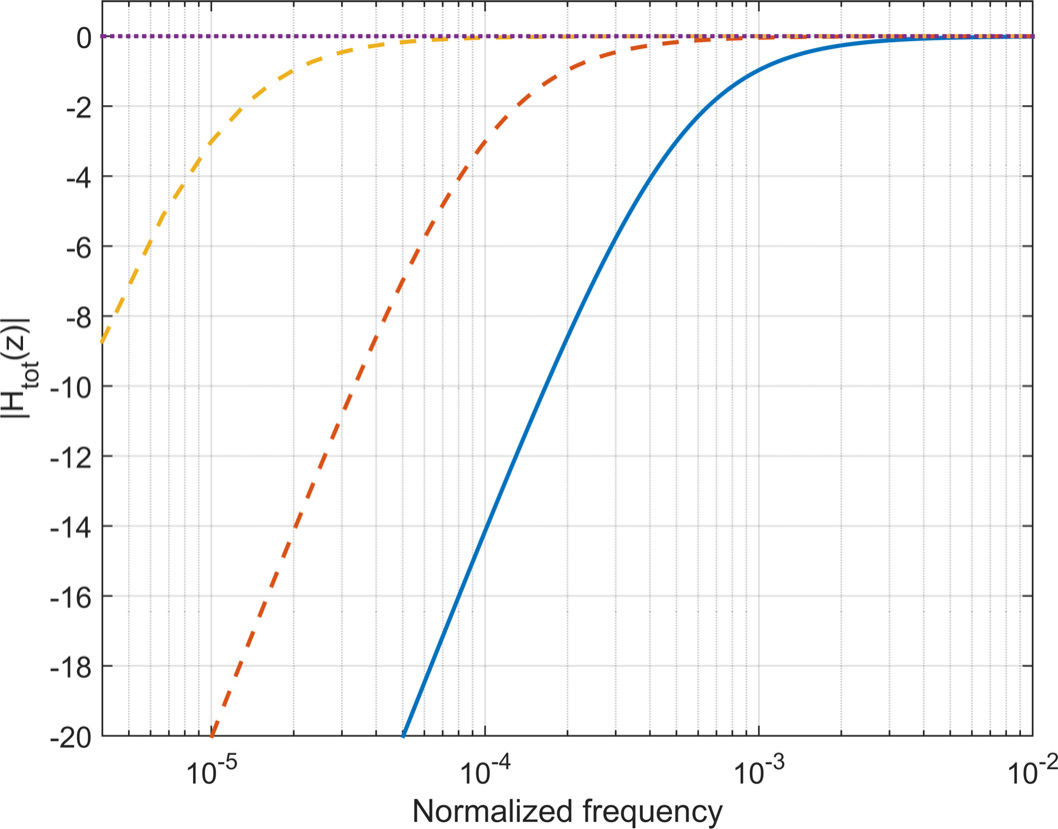
Transfer function. For increasing c>β, the transfer function is shifted to the left (dashed lines), lowering the cut-off frequency. For c = 1, the spectrum becomes flat (purple, dotted line). β is constantly 0.996863, c takes on the values of [β, 0.999372, 0.999937, 1.000000].

The outcome is that a stable inverting high-pass filter can be designed from a maximum gain specification. On Fig 1, the principle behind the iHPF is shown through transfer functions: the transfer function of the overall system, *H*_*tot*_(*z*) = *H*_1_(*z*)*H*_3_(*z*), is then a shifted version of the original HPF, H_1_(z). As c takes on values different from β, the transfer function moves along the frequency axis (see Fig 1). As c increases towards maximally 1, the transfer function is shifted to the left, giving a lower cut-off frequency. In the limiting case, where c = 1, the transfer function has been shifted infinitely to the left, yielding a flat transfer function (H(z) = 1) with a constant gain of unity and no phase changes (*i*.*e*. no changes are made to the signal waveform at all). If the value of c is lowered below β, the transfer function is correspondingly shifted to the right, resulting in a higher cut-off frequency for the overall system, H_tot_(z).

### Frequency mismatch

A digital AC-filter can be perfectly cancelled when the filter coefficients are known, since they are fixed. For an analog AC-filter, the components may vary from their prescribed values, leading to a difference in cut-off frequency compared to the intended value. Predicting the response for a first order high-pass filter is fairly easy. We know that the slope of the filter is 20 dB/decade for frequencies much smaller than the cut-off frequency. Closer to the cut-off, the attenuation is small and therefore the overall error (mismatch between attenuation and gain) will be smaller. The maximum respective minimum linear gain is the inverse of the factor, by which the cut-off frequency has been shifted from its intended value (see appendix A for further details). If the cut-off frequency has been changed by a factor of 1.05 (is 5% higher than the intended value), the maximal respective minimal gain will be 1.05^−1^ = 0.952, which in turn is approximately a five percent deviation.

### Test setup

Three datasets of anonymized ECG recordings from multiple studies and centers, recorded with a DC-coupling, were subjected to testing. Recordings from the same ECG-device constituted a class (see Table 1). To ensure consistency, exactly one minute from each recording was used, and the ECGs were standard 12-lead ECGs. This meant the exclusion of 1320 recordings (41%) that were shorter than one minute in length. An automatic lead-quality assessment system [20] was used to find the first minute of each recording with no lead-off. If no such entire minute was available within the first ten minutes, the recording was excluded (290 recordings, 9%). 29 recordings (1%) could not be read properly and were thus excluded. In total, 1620 recordings were included.

**Table 1:** Datasets. Overview of datasets used for the evaluation.

Class name	SwissAF [21], device 1	SwissAF [21], device 2	APACE [22], device 1	Total
Recordings available from the study	915	328	2016	3259
Less than a minute	307 (34%)	215 (66%)	798 (40%)	1320 (41%)
Could not be read	5 (1%)	2 (1%)	22 (1%)	29 (1%)
No suitable minute	173 (19%)	7 (2%)	110 (5%)	290 (9%)
Total excluded	485 (53%)	224 (68%)	930 (46%)	1639 (50%)
Total included	430 (47%)	104 (32%)	1088 (54%)	1620 (50%)

**Fig 2:**
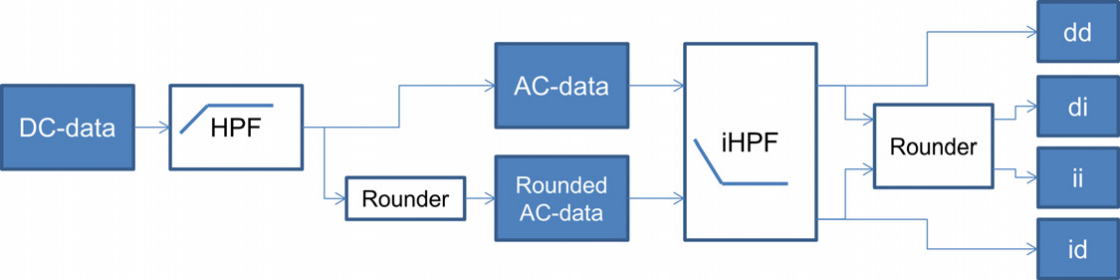
Test setup. The data flow for the test is shown. Labels “d” and “i” stand for “double precision” and “integer” respectively, indicating if rounding was included in the path or not, and when.

The recordings were stored in 20-bit-integer format, they have a resolution of 1 μV/LSB, and they have been sampled at 1 kHz, with an anti-aliasing filter having F_c_ = 387 Hz, and without any AC-coupling or other high-pass filter (CS-200 Excellence, Schiller AG, Baar, Switzerland). As shown on Fig 2, each digital ECG was first filtered by a first order HPF and afterwards by an iHPF. The cut-off frequency was set to 0.05Hz. Paths with rounding operations after each filtering were included, so that four cases appear for each iHPF tested.

To assess the maximum error, the effects from path “ii” are taken into consideration. The first rounder adds an error of maximally 0.5 LSB. This error is then amplified by the iHPF based on the frequency. To guarantee that the total error is maximally E LSB, the gain, 1/G, of the iHPF (corresponding to a gain, G, for the HPF) must be such that the 0.5 LSB maximal error from the first rounding is not multiplied to a level greater than or equal to E+0.5 LSB (5). This requirement ensures that the second rounding quantizes the error to maximally E LSB. This model assumes rounded input data, and that c = 1 for the iHPF. The model might also be valid for a finite M.

$$0.5*\frac{1}{G}<E+0.5$$ (5)

Given a maximum error of E LSB, the minimum gain can be found (6) for E∈N. The special case of G = 1 causes the first rounding–as well as the final result–to have zero error. The special case of E = 0.5 LSB requires the first rounding to produce an error of zero, which is only the case for G = 1.

$$G>\frac{1}{2E+1}$$ (6)

Assuming a first order HPF with cut-off frequency F_c_ = 0.05Hz, Table 2 shows the connection between maximal error, minimal gain (HPF), and the frequency at which this gain is found for a first order 0.05 Hz HPF. The maximal gain of the iHPF is the inverse of the second column.

**Table 2:** Maximum error versus gain.

Maximum error [LSB]	Minimum gain [V/V]	Minimum gain [dB]	Minimum frequency^a^ [Hz]	Maximum period^a^ [s]
0	1	0	0	Infinite
0.5	1	0	0	Infinite
1	1/3^b^	-9.54	0.0354	28.3
2	1/5^b^	-13.97	0.0250	40.0
5	1/11^b^	-20.82	0.0158	63.2
10	1/21^b^	-26.44	0.0112	89.4
25	1/51^b^	-34.15	0.0071	141.4

Requirements for a given maximum error (path “ii”)

^a^Using a HPF of type (1) with F_c_ = 0.05 Hz and F_s_ = 1000 Hz.

^b^Minimum not included in valid range.

Errors–defined as differences between the recovered DC-signals (“dd”, “di”, “id”, “ii”) and the input signal (“DC-data”)–were counted with a resolution of 0.05 LSB using the *histc()* function in MATLAB (MATLAB 8.5, The MathWorks, Inc., Natick, MA). RMS-values were also calculated for each output signal against its input signal with both signals having had their respective averages subtracted since the offset is of no interest and should not influence the morphology analysis.

## Results

**Fig 3:**
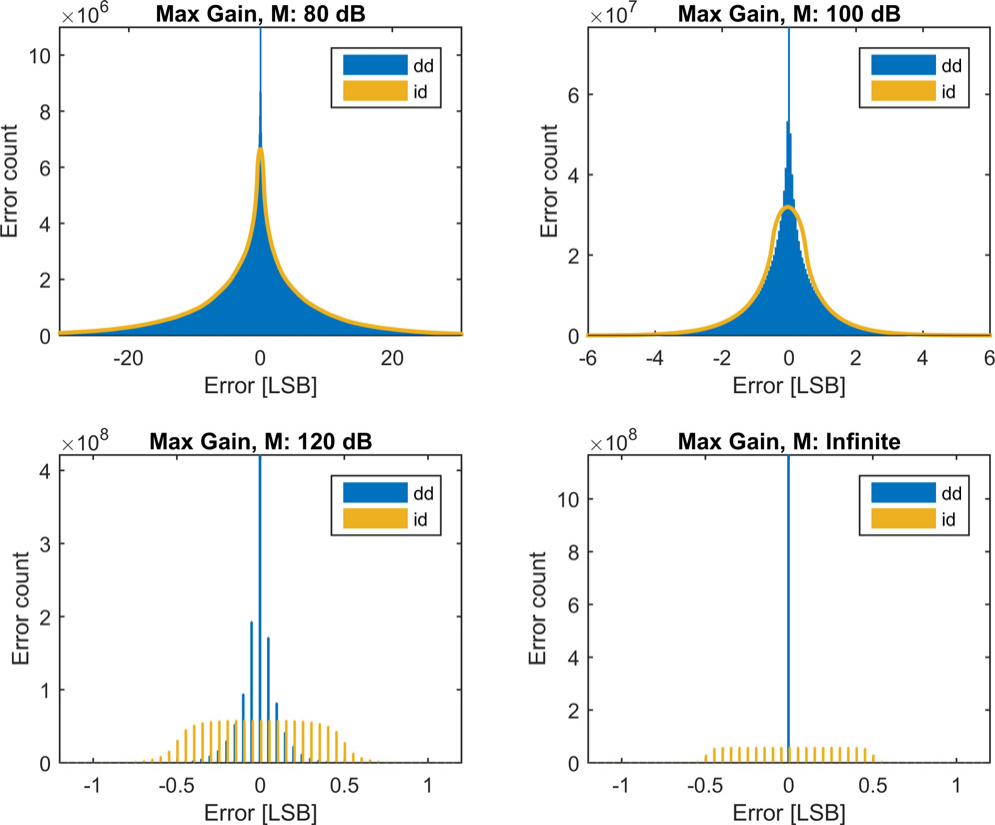
Histograms. Histograms of error amplitude (bin width of 0.05 LSB) for the selected iHPFs. Only paths without the second rounding have been included to make the graphs more readable. The effects of the final rounding can easily be derived from the graphs.

In Fig 3, histograms of the errors are plotted for paths “dd” and “id” for M equal to 80 dB, 100 dB, 120 dB and for the exact reconstruction (M tending to infinity). The results for the two remaining paths (Fig 2) can be found by moving each bar to the nearest integer.

**Fig 4:**
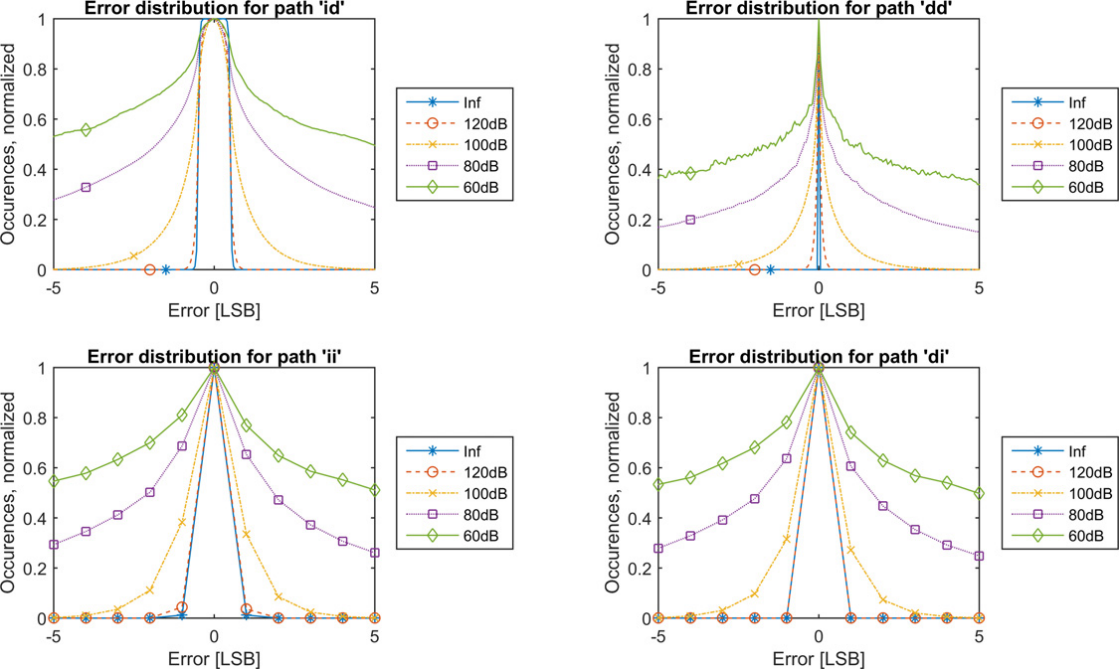
Histogram contours. For increasing DC-gain, M, the contours of the histograms (normalized by their maximum value) shrink towards the center, showing that the error produced is lowered.

On Fig 4, envelopes of the histograms have been depicted for various maximal DC-gains, M. These envelopes have been normalized against their maximum value for easier comparison. All paths from Fig 2 have been included.

**Fig 5:**
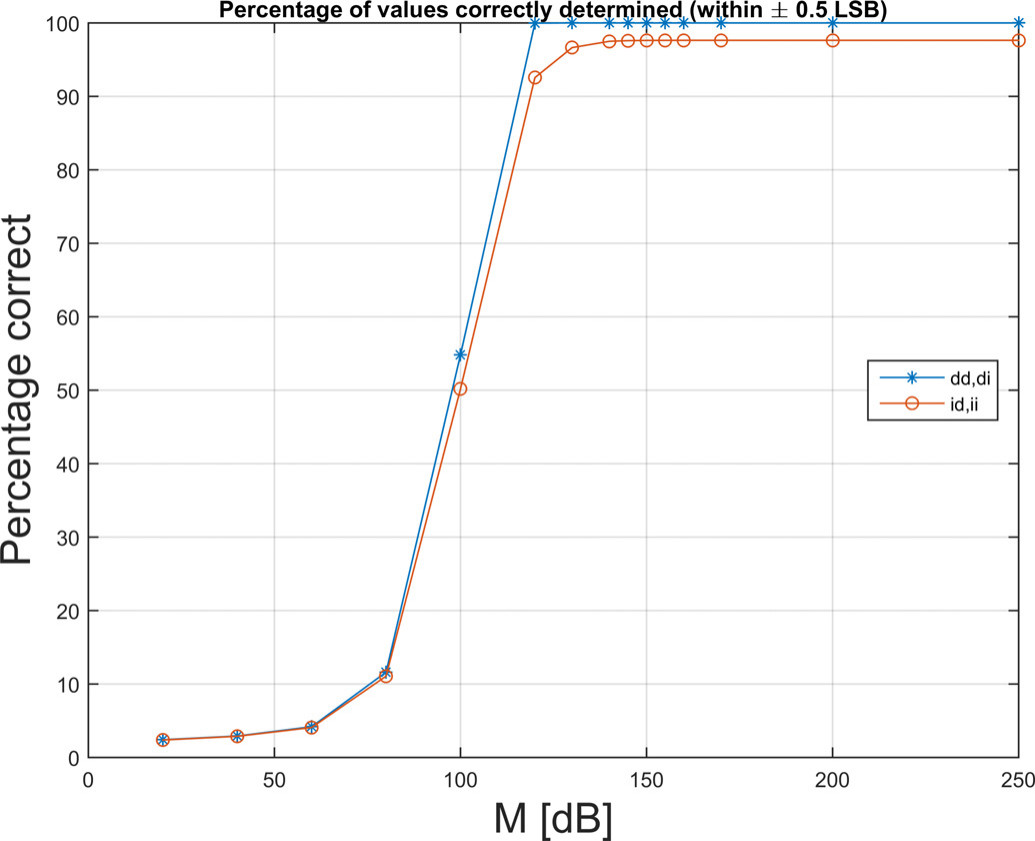
Exact reconstruction. For increasing DC-gain, M, the percentage of values that are estimated correctly (to a ±0.5 LSB level) increases towards 100%. From around 120 dB, all values are correctly restored when no initial rounding takes place. In case of rounding after the AC-coupling, 97.6% percent of the values can be correctly determined to within this level. Fig 4 shows the distribution of the errors.

The percentages of samples that are being bit-exactly recovered for various DC-gains, M, are depicted on Fig 5. With an initial rounding, not every sample can be recovered.

**Fig 6:**
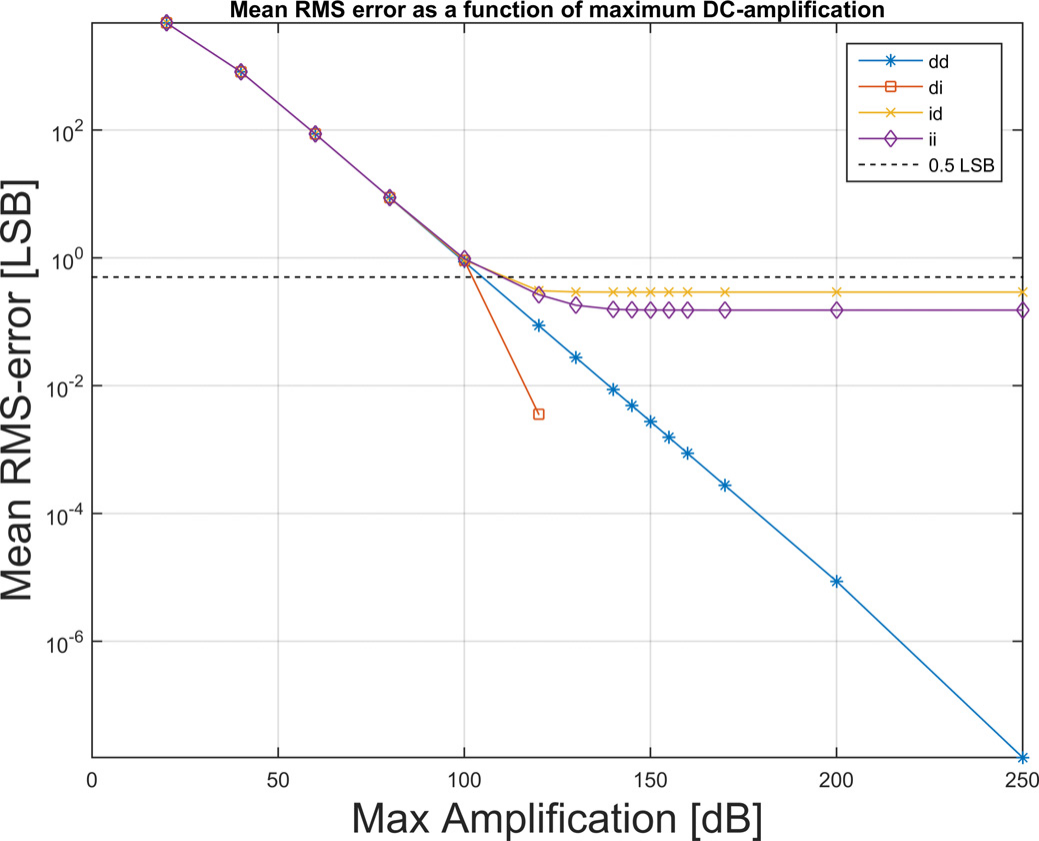
RMS-error. The mean RMS-error decreases for increasing DC-gain, M, here shown on a log-log plot. The mean RMS error for the perfect case (M→∞, path “dd”) was found to be 3.1*10^−8^ LSB. For M≥110 dB the RMS error is below 0.5 LSB for all paths. For M≥125 dB, no error is greater than ±0.5 LSB when no initial rounding takes place, and the error drops to zero for path ‘di’ (red squares) since the output is rounded to the correct integer value.

The RMS of the error is depicted on Fig 6 for all paths (Fig 2) and for increasing values of maximal DC-gain, M. For path “di”, the second rounding eliminates all errors smaller than ±0.5 LSB when the input data are integers only. Since for M≥125 dB all errors are within this limit, the RMS-value drops to zero, which cannot be shown on the logarithmic ordinate.

## Discussion

### Practical errors and filter strength (M)

Fig 3 shows how the errors become larger–they “spread out”–when the inverse filter becomes weaker. The practical errors, however, do not reach the level of the theoretical error in any case. Signal lengths of one minute could theoretically lead to errors of ±2 LSB (see Table 2) in case they contain the worst case combination of frequency and amplitude. In practice, resting ECG signals appear to be very far from the worst case and only very few samples are being rounded to a neighboring bit-value (2.4%).

A practical value for the maximal DC-gain, M, of the inverse filter should be selected based on the tolerated maximal error. To minimize the error, an M in the order of 125 dB may be suggested. An M of 100 dB ensures an error of less than 5 LSB, which–even for 5 μV/LSB–is within the strictest requirements for accuracy [3,4].

The white noise that is added by the rounding operation persists in the recovered waveforms, as it is clear from comparison of paths “id” (Fig 4, top left) and “dd” (Fig 4, top right). Since the input data are rounded, in most cases a final rounding operation will restore the bit-exact value when the filter is sufficiently strong. At most 2.4% of the data points will be estimated incorrectly (Fig 5). In this case, the error is 1 LSB (Fig 3, bottom row).

When operating with double precision only, it seems reasonable to choose as high an M as possible. As soon as rounding operations are introduced, the advantage from using a stronger inverting filter is lessened, and a finite DC-gain should be selected.

In case of no initial rounding, *i*.*e*. saving of double-precision data, Fig 5 shows how every single data point is restored to its bit-exact value for M≥125 dB, when the input data are integers and the final output are rounded.

### Perfect reconstruction

The efficiency of the ideal filter is only limited by the precision with which calculations are performed. Using double-precision arithmetic, the mean of the RMS-error for each signal (1 minute, 12 channels) was 3.1*10^−8^ LSB, which can be considered negligible. When the inverse filter is stabilized (by weakening the filter) the mean RMS-error increases. The blue curve (stars) on Fig 6 (“dd”) shows this behavior, and reveals that the RMS error is below 0.5 LSB for filter strengths (DC-gain) M≥110 dB for F_c_ = 0.05 Hz. From the same figure, it can be seen that for M≥125 dB, no single sample is reconstructed with an error greater than 0.5 LSB (red line, squares).

When an initial rounding takes place after the high-pass filtering, a noise is added which cannot be guaranteed to be removed. Therefore, there exists a lower limit for the RMS-error. As can be seen on Fig 6, this lower limit is below 0.5 LSB. The error distribution can be seen on Fig 3, and is limited to less than ±1 LSB for the ideal filter. For the stabilized filters, the histogram widens as the filter is weakened. Maximally ±1 LSB is achieved at least for strengths M≥120 dB.

Throughout this work, we have assumed that the offset cannot be recovered, and this has therefore manually been adjusted for, so that the offset plays no role in the evaluation. Not being able to recover the offset is certainly the case for an analog high-pass filter when the recording is not started until all electrodes have been attached.

### Analog and digital high-pass filters

All simulations have been carried out with a digital high-pass filter. The considerations cannot be fully applied to the case of an analog filter. For one, the first order digital and analog high-pass filters are not identical. Their responses are similar for F_c_<<F_s_, but they become increasingly different for higher F_c_/F_s_ ratio. For F_c_ = 0.05 Hz and F_s_ = 1 kHz, the digital and analog versions are considered equivalent.

When an analog high-pass filter is used, the output of the ADC is not high-pass filtered. This means that if the ADC adds a DC-offset, this would persist in the signal. Such a DC-offset would then be amplified by the inverting filter, which could lead to instability if the offset is not subtracted beforehand.

The use of digital high-pass filters ensures that we know the exact cut-off frequency of the filter. If an analog filter had been used, the same precision could not be guaranteed. If an ideal analog filter could be used, one may find tiny deviations compared to an equivalent digital filter because there is no rounding before the high-pass filter. This would also mean that the RMS-error of path “di” would not drop to zero as it does. Instead, on Fig 6, the error curves of paths “di” and “ii” would include white-noise rounding-error as is yet the case for path “id”. For path “ii”, two such errors would be added.

### Limitations

The results in this article are applicable to a maximum signal length of one minute. This more than covers the standard resting ECG of typically 10 seconds, which is the most common use of ECG. For longer recordings, such as exercise ECG and Holter ECG, the method has not been validated in practice. Our future work may include verification of the method for these kinds of ECG recordings.

The testing has been performed with the 0.05 Hz filter suggested by the current guidelines for diagnostic ECGs [4]. For other use cases of ECG, such as monitoring devices, other standards apply that allow for higher cut-off frequencies. A higher cut-off frequency would mean that more signal contents would be suppressed to a level from which it cannot be recovered. The right half of Table 2 would have to be re-calculated for different cut-off frequencies. In practice, unpublished results indicate that even for f_c_ = 0.3Hz, all errors are below 2 LSB for these datasets.

### Pulse test

**Fig 7:**
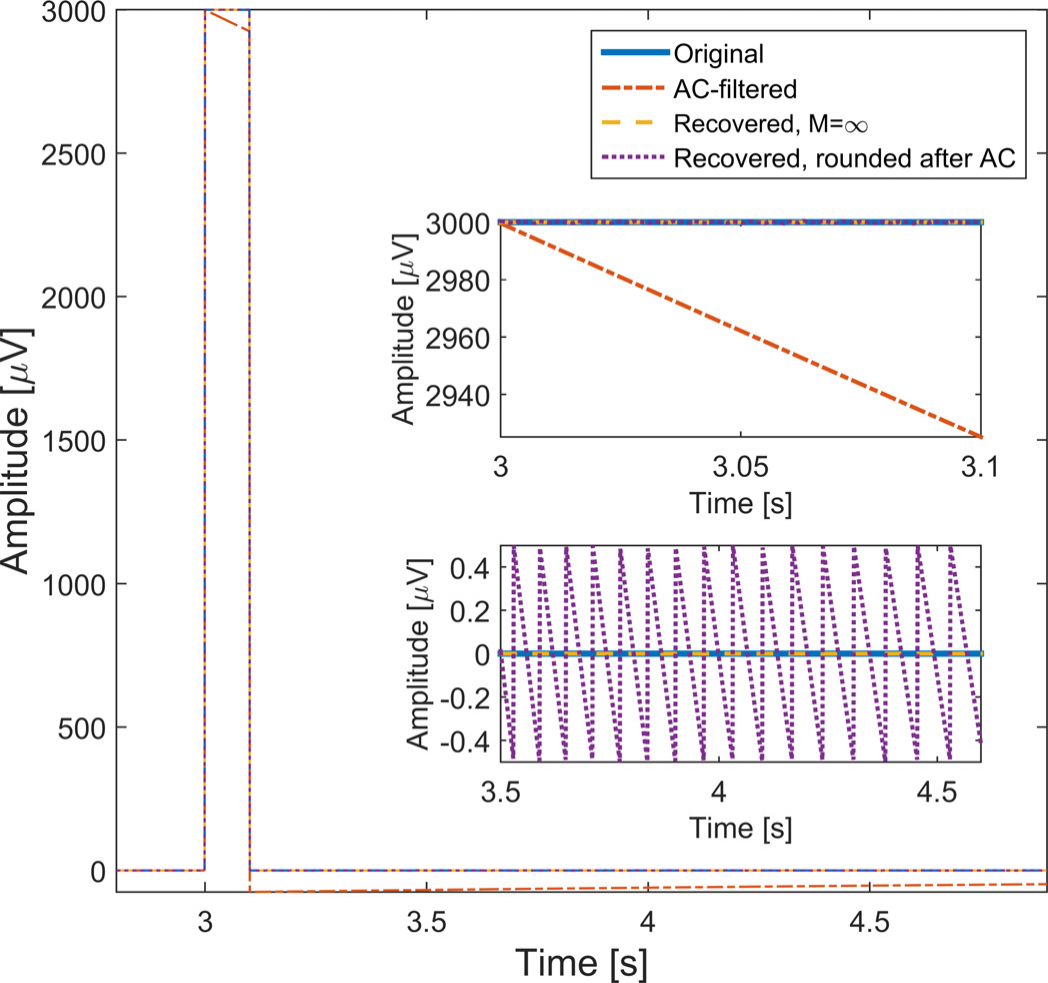
Pulse filtering. The filtering of the 300 μV·s pulse (1μV/bit) followed by reconstruction with the ideal inverse filter reveals the successful design of the inversing filter. With an initial rounding after the AC-coupling, the output error signal is within ±0.5 μV as expected (see inset). The offset that can be seen for the AC-signal is exactly the effect which caused Berson *et al*. [1] to raise the alarm. This offset is completely removed by the inverse filter.

As a second validation, the 300 μV·s testing pulse (3mV for 100 ms) [3,4] was fed through the test setup with no final rounding (paths “dd” and “id”). The results of this test are depicted on Fig 7. When no rounding takes place, no error occurs. The white-noise error from the quantizing persists just as was the case for real ECG data. The successful recreation of this pulse, which has traditionally been used for ECG testing, is a positive sign for the method presented.

## Conclusions and Outlook

We have shown the successful recovery of original “DC-coupled” ECG signals from signals that had been AC-coupled with a digital high-pass filter. This was done using an inverse high-pass filter. Even with rounding operations, the error is maximally ±1 LSB and this error occurs at most for 2.4% of the samples. If stability of the inverse filter is an issue, a weaker alternative can be used. A filter with DC-gain M = 120 dB performs similarly for a cut-off frequency of 0.05 Hz for the high-pass filter.

As we can now reconstruct the original “DC-coupled” ECG signals from AC-coupled ones, we will next investigate the influence of the AC-coupling onto the signal morphology and its consequence on clinical interpretation. We already know that the AC-coupling is artificially generating ST-depressions and–elevations, mimicking acute or chronic ischemic heart disease. These two diseases are the main target diagnoses of the ECG in daily use today. With access to large existing clinical databases, the proposed filter enables the usage of 0.05 Hz AC-coupled digital signals in answering this question in full details, since the DC-signals can be obtained for comparison.

## Appendix A (Digital Simulation of First Order AC-Coupling)

A general first order digital high-pass filter (here denoted by H_AC_(z)) is given by Eq 7. It has an overall gain of α and a cut-off frequency that depends on β.

$$H_{AC}\left(z\right)=\frac{\alpha \left(z-1\right)}{z-\beta }=\frac{\alpha \left(1-z^{-1}\right)}{1-\beta z^{-1}}$$ (7)

If we require the filter to have a gain of unity in the pass band, the value of α becomes dependent on β (Eq 8)

$$|H_{AC}\left(z=-1\right)|=\left|\frac{\alpha \left(-1-1\right)}{-1-\beta }\right|=1$$ (8)

The linear dependency allows for a rewriting of Eq 7 into Eq 9:

$$H_{AC}\left(z\right)=\frac{1+\beta }{2}\frac{z-1}{z-\beta }$$ (9)

If we furthermore define the cut-off frequency as the frequency at which the transfer function has an absolute value of $\frac{\sqrt{2}}{2}$ (-3dB), the β-coefficient can be directly determined based on the desired normalized cut-off frequency $f_{c}=\frac{F_{c}}{F_{s}}$, whereby F_c_ is the cut-off frequency and F_s_ is the sampling frequency, both with identical units.

$$\left|H_{AC}\left(e^{j2\pi f_{c}}\right)\right|=\left|\frac{1+\beta }{2}\frac{e^{j2\pi f_{c}}-1}{e^{j2\pi f_{c}}-\beta }\right|=\frac{\sqrt{2}}{2}$$ (10)

$$\beta =\frac{1-\text{sin}\left(2\pi f_{c}\right)}{\text{cos}\left(2\pi f_{c}\right)},\;f_{c}\in [0;\frac{1}{2}[$$ (11)

Note also that the HPF reduces to a constant of unity gain (which makes no changes to the signal) when f_c_ = 0, as one would expect.

For H_AC_(z), given a value of β, the normalized cut-off frequency can be determined Eq 12. By multiplication of f_c_ with the sampling frequency, F_s_ in Hz, the cut-off frequency, F_c_ is returned in units of Hz.

$$f_{c}=\frac{\text{acos}\left(\frac{2\beta }{\beta^{2}+1}\right)}{2\pi }$$ (12)

For F_c_<<F_s_, the main difference between a digital and an analog filter is the precision with which the filtering takes place. The coefficients of the digital filter are much more precise than the components that constitute the analog filter. Assuming an error factor of δ, such that the real cut-off frequency, F_c,real_, equals the intended frequency, F_c,intended_, times delta, the maximum error in Decibels can be calculated (using the first order slope of 20 dB/decade):

$$e_{dB}=-20\left({\text{log}}_{10}\left(F_{c,real}\right)-{\text{log}}_{10}\left(F_{c,intended}\right)\right)=-20\;{\text{log}}_{10}\left(\frac{F_{c,intended}\delta }{F_{c,intended}}\right)=-20\;{\text{log}}_{10}\left(\delta \right)$$ (13)

As an example, for an error of 10%, we have δ = 1.1 and:

$$e_{dB}=-20\;{\text{log}}_{10}\left(1.1\right)=-0.828\;dB$$ (14)

The error used in Eq 14 corresponds to a linear gain of 1.1^−1^ = 0.909.
